# Human Capital and Reemployment Success: The Role of Cognitive Abilities and Personality

**DOI:** 10.3390/jintelligence5010009

**Published:** 2017-03-22

**Authors:** Timo Gnambs

**Affiliations:** Leibniz Institute for Educational Trajectories, 96047 Bamberg, Germany; timo.gnambs@lifbi.de; Tel.: +49-951-863-3420

**Keywords:** intelligence, competence, personality, unemployment, job search

## Abstract

Involuntary periods of unemployment represent major negative experiences for many individuals. Therefore, it is important to identify factors determining the speed job seekers are able to find new employment. The present study focused on cognitive and non-cognitive abilities of job seekers that determine their reemployment success. A sample of German adults (*N* = 1366) reported on their employment histories over the course of six years and provided measures on their fluid and crystallized intelligence, mathematical and reading competence, and the Big Five of personality. Proportional hazard regression analyses modeled the conditional probability of finding a new job at a given time dependent on the cognitive and personality scores. The results showed that fluid and crystallized intelligence as well as reading competence increased the probability of reemployment. Moreover, emotionally stable job seekers had higher odds of finding new employment. Other personality traits of the Big Five were less relevant for reemployment success. Finally, crystallized intelligence and emotional stability exhibited unique predictive power after controlling for the other traits and showed incremental effects with regard to age, education, and job type. These findings highlight that stable individual differences have a systematic, albeit rather small, effect on unemployment durations.

## 1. Introduction

The loss of paid work and ensuing periods of unemployment present severe negative repercussions for not only the person concerned but also their families and the society as a whole. These adverse effects go well beyond a mere loss of material prosperity. Enduring periods of unemployment deteriorate physiological and psychological well-being and, among others, result in more depressive symptoms and higher suicide rates [1,2,3]. Therefore, understanding the process of job search and identifying relevant factors explaining the probability of finding a new job represents a crucial concern for those affected by unemployment’s consequences. The present study focused on the human capital of job seekers to identify cognitive and non-cognitive factors predicting their reemployment success. Adopting a longitudinal perspective, the employment histories of unemployed respondents were examined to identify the unique effects of domain-general intelligence, domain-specific competences, and enduring personality traits on the time until successful reemployment.

### 1.1. Human Capital and Reemployment Success

The speed that job seekers manage to find an adequate job matching one’s interests, skills, and work expectations (e.g., with regard to wage) after periods of involuntary unemployment is determined by various factors (for a review, see [3]). Among others, reemployment success is influenced by current labor market needs, (e.g., regional unemployment rates), a job seeker’s economic need to work (e.g., debts or dependent children), an individual’s job search intensity, and, importantly, also his or her human capital [4]. Rather enduring personal characteristics that reflect who one is and what one knows have a substantial impact on an individual’s employability [5]. Human capital subsumes several work-related individual differences such as job experience, education, or age, but also various psychological traits (e.g., intelligence, personality) [3]. Economic and sociological studies, for the most part, focused on sociodemographic differences that shape job seekers’ success in finding adequate employment. For example, this research showed that older job seekers experience significantly longer unemployment periods [6], whereas highly educated individuals tend to have greater reemployment success [7]. In contrast, research on psychological characteristics in the form of cognitive skills (e.g., intelligence) and personality (e.g., with regard to the Big Five) that might shape job seekers’ success in finding adequate employment has been less thoroughly scrutinized [3,8].

Despite a substantial body of research on job-search behavior and its antecedents [9,10], few studies addressed the relevance of cognitive abilities for reemployment success. Several prospective studies identified intelligence measured in childhood or adolescence as an influential predictor of unemployment in later life [11,12,13]. For example, representative data from Sweden highlighted that cognitive abilities assessed in youth that increased by one standard deviation corresponded to an increase in the probability of receiving unemployment benefits in adulthood by about 2 percent [14]. Similar results were identified in the United States: Herrenstein and Murray [13] reported that intelligence scores obtained in adolescence were significantly associated with the probability of being out of the labor force ten years later. Together, these findings indicate that cognitive factors can explain the likelihood of unemployment spells over an individual’s life course. However, these results do not show whether cognitive skills might also be relevant for the speed of finding a new job after becoming unemployed. Empirical evidence from personnel selection programs indicate that intelligence represents the most important predictor of job performance [15] and, thus, represents an important criterion for human resource professionals for selecting job candidates. Thus, it is conceivable that cognitive factors might also determine the reemployment success of job seekers. Indirect support for this assumption comes from studies linking reemployment success to schooling outcomes [8,16]: job seekers with a higher education were more likely to find reemployment faster than less educated individuals. Given the strong association between intelligence and academic performance [17], it can be assumed that cognitive skills might be similarly relevant for finding a new job.

A full understanding of psychological characteristics determining reemployment success needs to incorporate non-cognitive skills alongside cognitive factors [18]. Important labor market outcomes such as occupational attainment are not only affected by cognitive abilities but also by various personality traits [19,20,21]. Even reemployment success is partly influenced by basic traits of personality [14,22]. Meta-analytic summaries [8], albeit based on only two to four studies, reported significant negative associations between the Big Five of personality and the duration of unemployment. Higher levels of extraversion, conscientiousness, agreeableness, and openness were associated with significantly shorter unemployment periods. However, with correlations of about *r* = −0.10, the respective effects were rather small. So far, it is not clear whether these correlations reflect a unique effect of personality on reemployment success or, rather, represent an indirect effect of basic cognitive abilities. Many personality traits exhibit systematic associations with domain-independent intelligence and domain-specific competences [18,23,24]. For example, reasoning correlates at about *r* = 0.25 with openness to experiences and at *r* = −0.14 with conscientiousness [25]. Typically, cognitive abilities account for about 5% to 10% of the variance in personality [26]. Given the interdependence of cognitive and non-cognitive skills [18], it is unclear whether personality traits explain incremental variance in reemployment success beyond cognitive skills.

### 1.2. The Present Study

Human factors play an important role in finding a new job. However, few studies examined the relevance of job seekers’ psychological characteristics in the form of cognitive skills (e.g., intelligence, competence) and personality (e.g., Big Five) for reemployment success. Thus, the present study answers repeated calls [19,20] to contrast cognitive and non-cognitive factors to identify the unique predictive power of intelligence and personality traits for important labor market outcomes. Moreover, given the documented returns of educational investments on economic success [8,16], the study also acknowledged domain-specific competences as a specific form of cognitive skills that are gained during one’s school and college education. Thus, the present study on a representative sample of German adults aims at disentangling the unique effects of domain-independent intelligence from acquired competences and enduring personality traits.

## 2. Materials and Methods 

### 2.1. Sample and Procedure

The participants were drawn from the longitudinal National Educational Panel Study (NEPS) that followed a representative sample of German adults over the course of several years [27]. The surveys and cognitive tests were administered at the respondents’ private homes by trained interviewers from a professional survey institute. Further information on the sampling procedure, the data collection process, and the interviewer selection and training is summarized on the project website (http://www.neps-data.de). The present analyses focus on *N* = 1366 individuals (54% women) forming part of the active labor force between 18 and 60 years of age (*M* = 41.08, *SD* = 11.14) that exhibited at least one unemployment spell between 2010 and 2015. The sample was well-educated, with about 53% having university entrance qualifications corresponding to levels 3 to 4 of the International Standard Classification of Educations (ISCED) version 1997 [28] and about 39% having finished a university education (equivalent to ISCED levels 5 or higher). The most recent employment of the respondents spanned various fields including blue-collar (30%) and white-collar professions (70%). The International Socio-Economic Index [29] that reflects an individual’s economic and social position based on his or her most recent occupation (range: 0 to 100) was *M* = 43.89 (*SD* = 17.42).

### 2.2. Instruments

#### 2.2.1. Time to Reemployment

All respondents provided full occupational histories across their life courses including their employment and unemployment spells. For each new unemployment spell starting between 2010 and 2015, I calculated the duration (in weeks) until reemployment, that is, the number of weeks until an unemployed respondent found a new job. I only considered involuntary unemployment spells characterized by respondents that were out of paid work and were actively seeking paid employment. Thus, non-employment spells due to, for example, maternity leave, sick leave, or an educational hiatus were not considered. Each respondent exhibited between 1 and 9 unemployment spells (*Mdn* = 1) within the examined time frame, totaling in 1973 unemployment spells for the entire sample. About 68% percent of all included spells ended in reemployment, whereas the remaining spells were still ongoing at the end of 2015 (i.e., the final measurement occasion of this study). The mean time until reemployment was *M* = 25.64 weeks (*SD* = 27.58).

#### 2.2.2. Intelligence

Fluid and crystallized intelligence were operationalized with two tests measuring reasoning and receptive vocabulary that were administered in 2014. *Fluid intelligence* was measured with a matrices test including 12 items [30,31]. For each item, respondents had to identify a basic logical rule and select a geometrical element that followed this rule. The sum score of correctly solved items represented the respondents’ reasoning abilities as an indicator of their fluid intelligence. On average, the participants correctly solved *M* = 8.33 (*SD* = 2.80) items. The omega hierarchical reliability was ω_h_ = 0.88 [32]. *Crystallized intelligence* was measured with the Peabody Picture Vocabulary Test [33,34] that included 89 items. For each item, the respondents had to select one out of four pictures that corresponded to a spoken word. The number of correctly answered items represented the measure of receptive vocabulary as an indicator of crystallized intelligence. The average score of the respondents was *M* = 71.84 (*SD* = 10.97) and the reliability was ω_h_ = 0.97.

#### 2.2.3. Competences

Domain-specific competences were measured in 2010 with achievement tests focusing on mathematics and reading. The adopted theoretical frameworks for these tests are described in [35,36]. Both tests were scaled using models of item response theory. Competence scores were estimated as weighted maximum likelihood estimates (WLE; [37]). The WLE reliabilities were good with 0.78 for the mathematical test and 0.72 for the reading test, respectively. Detailed psychometric properties of the administered tests are reported in [38,39].

#### 2.2.4. Personality

The five basic traits of personality—openness, conscientiousness, extraversion, agreeableness, and emotional stability—were assessed with a short version of the Big Five Inventory [40] that measured each trait with two or three items. Each item was accompanied by a five-point response scale from *fully disagree* (1) to *fully agree* (5). Four scales exhibited acceptable reliabilities with ω_h_ around 0.70 (see Table 1). However, agreeableness was inadequately measured with ω_h_ = 0.45.

#### 2.2.5. Control Variables

Several control variables were acknowledged in the analyses, including the respondent’s sex (coded 0 for women and 1 for men), age (in years), and the years of education [41]. Moreover, respondents’ most recent occupations were coded according to the International Standard Classification of Occupations (ISCO). Following the methodology adopted in the Programme for International Student Assessment [42], I grouped these occupations into blue-collar jobs (ISCO 6 and higher; coded 0) and white-collar jobs (ISCO 1 to 5; coded 1).

### 2.3. Statistical Analyses

The effects of intelligence, competence, and personality on the time until reemployment were examined with proportional hazard regression analyses [43] that modeled the conditional probability of reemployment (i.e., finding a new job) at a given time interval as the dependent variable. These analyses used the Kaplan–Meier estimator [44] that also acknowledges right-censored data (i.e., ongoing unemployment spells). Because some respondents experienced multiple unemployment spells during the examined period, these dependencies were acknowledged by computing robust variances using the Huber–White sandwich estimator [45]. Moreover, given that some respondents exhibited missing values on one or more variables, these analyses were based on multiple imputations, where missing values were imputed 50 times using predictive mean matching [46]. Effect sizes were evaluated in line with conventional standards [47] with odds ratios (OR) of 1.22, 1.86, and 3.00 indicating small, medium and large effects, respectively. To examine the unique and incremental effects of intelligence, competence, and personality with regard to the control variables, a two-step strategy was adopted [48]. First, the bivariate effects for each domain were studied without acknowledging any covariates. Then, the control variables were included to derive the incremental effects of each domain beyond age, educational level, and job type. Age was modeled as a time-varying variable, whereas educational level and job type were included as time-invariant predictors. Finally, an omnibus model was estimated that included all variables in a single model to derive the partial effect for each predictor controlling for the other. Different models were compared using the Bayesian Information Criterion (BIC; [49]) that indicates a better fit at smaller values.

### 2.4. Statistical Software and Open Data

All analyses were conducted in *R* version 3.3.2 [50] with the *survival* package version 2.40 [51] and *mice* version 2.30 [52]. The raw data is available at http://www.neps-data.de.

## 3. Results

**Figure 1:**
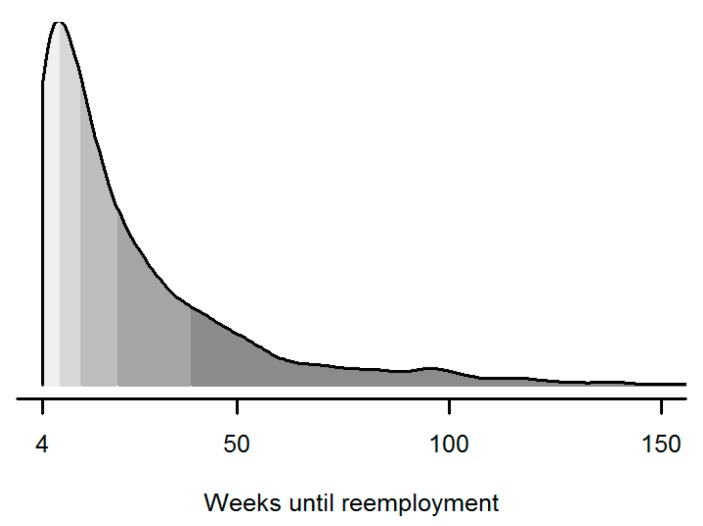
Distribution of weeks until reemployment. Each shade represents 20 percent of the sample.

The time to reemployment exhibited a rather skewed distribution (see Figure 1). About 25 percent of the respondents found a new job within little more than two months and 50 percent within four months. After six months, nearly 65 percent of the sample was reemployed, whereas the rest of the sample took a median of about eleven months until returning into paid employment. The average time to reemployment was not significantly different for blue-collar (*M* = 27.90, *SD* = 33.06) and white-collar workers (*M* = 24.68, *SD* = 24.86), *t*(*df* = 598.13) = 1.75, *p* = 0.08, *d* = 0.12. However, women (*M* = 27.15, *SD* = 27.07) took significantly, *t*(*df* = 1307.40) = 2.14, *p* = 0.03, *d* = 0.12, longer than men (*M* = 23.92, *SD* = 28.07) to find a new job, as did older respondents aged 50 years or above (*M* = 33.12, *SD* = 32.79) as compared to younger individuals (*M* = 23.57, *SD* = 25.60), *t*(*df* = 392.66) = −4.60, *p* < 0.001, *d* = −0.35.

Influential respondent characteristics determining the time to find a new job were examined by regressing the time to reemployment on the respondents’ fluid and crystallized intelligence, mathematical and reading competence, the Big Five of personality, and several control variables. Preliminary analyses indicated that the respondents’ sex violated the proportional odds assumption of the Cox [43] regression model. Therefore, the analyses were stratified by sex resulting in different baseline hazard functions for men and women but identical regression coefficients of the included predictors across sexes.

In the first step, the unique effects of intelligence, competence, and personality were studied without considering age, education, and job type as control variables. The results of these analyses are summarized in Table 2 (Model 1). Regarding individual differences in basic cognitive abilities, the odds of finding a new job at a given time interval significantly (*p* < 0.001) increased by 15 percent (OR = 1.15) for each standard deviation increase in fluid intelligence and by 6% for a standard deviation increase in crystallized intelligence (OR = 1.06, *p* = 0.03). Similarly, reading competences increased the probability of reemployment by about 14% (OR = 1.14, *p* = 0.004). In contrast, mathematical competences (OR = 1.06, *p* = 0.14) were not significantly associated with the odds of finding a new job. Finally, regarding the five personality traits only emotional stability was significantly (*p* = 0.02) associated with the odds of finding a new job; one standard deviation increase in emotional stability corresponded to an increase in the odds of reemployment of about 7 percent (OR = 1.07). Thus, fluid and crystallized intelligence, reading competence, and emotional stability independently predicted the examined hazard rates of reemployment.

In the second step, age, education, and job type were added to these regression models to study the incremental contributions of intelligence, competence, and personality with regard to these control variables (Model 2 in Table 2). These analyses failed to identify incremental effects of fluid intelligence (OR = 1.03, *p* = 0.20) or reading competence (OR = 1.07, *p* = 0.09) on the odds of reemployment. In contrast, crystallized intelligence (OR = 1.09, *p* = 0.01) and emotional stability (OR = 1.09, *p* = 0.004) remained significant predictors of the probability of finding a new job. Thus, education and job type accounted for the effects of fluid intelligence and reading competence, whereas crystallized intelligence and emotional stability exhibited unique associations with reemployment probability beyond these sociodemographic characteristics.

**Figure 2:**
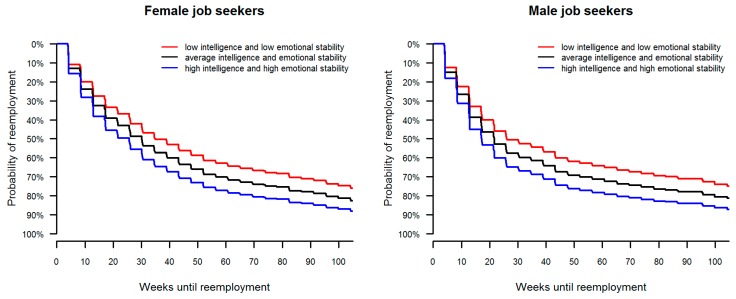
Estimated survival functions for time until reemployment by low (*M* − 1 *SD*) and high (*M* + 1 *SD*) crystallized intelligence and emotional stability for female and male job seekers.

Finally, these effects were replicated in an omnibus analysis that combined intelligence, competence, and personality into a single model (see Table 3). Again, crystallized intelligence (OR = 1.08, *p* = 0.03) and emotional stability (OR = 1.09, *p* = 0.01) showed unique effects on the odds of finding a new job, even after controlling for individual differences in fluid intelligence, competences, personality, and the three control variables. The respective effects are summarized in Figure 2. For respondents either high in crystallized intelligence or high in emotional stability, the odds of reemployment gradually increased with the unemployment duration. On average, the difference in the odds of reemployment between high (*M* + 1 *SD*) or low (*M* − 1 *SD*) levels on the respective traits was about 10 percentage points.

## 4. Discussion

In the wake of the recent global economic crisis, many countries recorded a sharp rise in unemployment rates, thereby giving renewed relevance to investigate predictors of reemployment. The central objective of the present study was to scrutinize the employment histories of a sample of unemployed adults to identify psychological characteristics that might explain the time until they found a new job. The presented results allow for three central conclusions. First, fluid and crystallized intelligence predicted the time to reemployment. Higher levels of intelligence resulted in greater reemployment success. However, the effect of reasoning was fully mediated by the job seekers’ education and job type. Fluid intelligence did not exhibit a unique predictive power beyond these sociodemographic characteristics. In contrast, general verbal competencies increased the probability of finding a new job, even after controlling for these sociodemographic differences. Thus, verbal skills seem to help job seekers to present themselves favorably during job interviews and, thus, contribute to their reemployment success [53]. Second, higher levels of reading competence predicted shorter unemployment periods. This might reflect the demands of the modern information age that, for many jobs, places increasing importance on written texts and, thus, text comprehension. Many work tasks (particularly in white-collar professions) require reading skills to quickly identify relevant information from, for example, work reports and derived conclusions from these texts. If reading skills represent valued competences for employees (see [54] for monetary returns of competences), this might increase job seekers’ chance to find reemployment. However, competences are primarily acquired in school and college. Therefore, they did not exhibit incremental effects beyond the number of years in education. Educational accomplishments in the form of higher academic degrees have been shown to predict the duration of unemployment periods [8]; this effect also replicated in the present study. Thus, educational levels seemed to fully mediate potential effects of competences on reemployment success. Indeed, a path model estimating the implied mediation effect in M*plus* 7 [55] revealed that the number of years in education exhibited a significant indirect effect on unemployment durations via reading competence, *B* = 0.03, *SE* = 0.01, *OR* = 1.03, *p* = 0.05, but not via mathematical competence, *B* = 0.01, *SE* = 0.01, *OR* = 1.01, *p* = 0.40. Third, personality explained unemployment durations beyond cognitive skills. These results contribute to the current discussion on the incremental effects of personality traits on economic outcomes [25]. As has been shown elsewhere [18], the Big Five of personality exhibit systematic associations with cognitive abilities. The most pronounced effects in this study were observed for conscientiousness that correlated negatively with domain-independent intelligence as well as domain-dependent competencies (see [21,23] for similar results). However, despite these associations emotional stability showed a unique predictive power on the time to reemployment. Thus, self-conscious individuals that are not easily frustrated by challenging situations were more likely to find a new job faster. This effect falls in line with previous findings that showed a higher probability of unemployment for individuals with mental disorders or maladaptive behaviors in youths [14,56].

Taken together, the study identified systematic effects of cognitive and non-cognitive factors with the time to reemployment. According to prevalent standards [48], the respective effects of crystallized intelligence and emotional stability, albeit significant, were rather small and can be considered negligible in size. One standard deviation increase in either intelligence or personality increased the odds of finding a new job by only about eight percent. However, on the individual level, both of these negligible differences can accumulate to noticeable differences in the odds of finding a new job. As presented in Figure 2, for respondents high in crystallized intelligence and high in emotional stability, the odds of reemployment at, for example, 20 weeks were at about 50%, whereas the respective odds fell at about 40% for respondents low (*M* − 1 *SD*) on these traits. Thus, certain psychological profiles can increase the chance of finding a new job at a given time interval.

The limitations of the present study point at intriguing opportunities for future research. For one, the study considered a rather short period of only six years. It might be informative to investigate longer periods of unemployment to examine whether the identified effects can be replicated for the long-term unemployed. Furthermore, the presented analyses were stratified by sex because of different baseline hazard rates for men and women. This might reflect the well-known effects of household composition on female labor participation [57,58]. The decision of many women to assume paid employment strongly depends on the presence of an additional earner in the household [59]. Thus, the present research should be extended in future studies by controlling for income effects of a second earner to study sex differences in reemployment success. Future research is also encouraged to examine the effectiveness of job-search programs for different subgroups of individuals. Thus, it might be conceivable that intervention programs aimed at boosting self-presentation or stress-managing skills might be more effective for job seekers low in emotional stability, whereas those with high emotional stability might profit more from trainings of specific job search skills [10]. Finally, the present study was limited to one specific aspect of reemployment success, namely, the time to reemployment. Future studies could extend these findings by addressing more qualitative components of reemployment success such as the match between a job seeker’s job expectations and her or his realized job conditions. Individual differences, particularly in personality traits, might explain why some individuals might be content with the “first best” job, whereas others are more selective in their job choice.

## 5. Conclusions

Overall, the present study showed that the time to reemployment was systematically associated with cognitive and non-cognitive factors. Higher levels of crystallized intelligence and emotional stability resulted in slightly shorter unemployment periods and a higher probability of finding a new job. However, the respective effects were small and explained few individual differences in unemployment durations beyond educational levels and job type.

## Figures and Tables

**Table 1:** Means, standard deviations, and correlations between study variables.

	Study Variables			Correlations											
		*M*	*SD*	1.	2.	3.	4.	5.	6.	7.	8.	9.	10.	11.	12.
1.	Fluid intelligence	8.27	2.80	*0.88*											
2.	Crystallized intelligence	71.39	11.00	0.43 *	*0.97*										
3.	Mathematical competence	−0.23	1.43	0.53 *	0.48 *	*0.78*									
4.	Reading competence	−0.27	1.42	0.47 *	0.55 *	0.62 *	*0.72*								
5.	Openness	3.59	0.96	0.09 *	0.22 *	0.07 *	0.16 *	*0.70*							
6.	Conscientiousness	4.03	0.75	−0.25 *	−0.20 *	−0.26 *	−0.22 *	0.08 *	*0.68*						
7.	Extraversion	3.41	0.97	0.02	0.02	−0.06 *	0.02	0.18 *	0.11 *	*0.69*					
8.	Agreeableness	3.58	0.66	−0.07 *	−0.11 *	−0.07 *	−0.04 *	0.11 *	0.15 *	0.07 *	*0.45*				
9.	Emotional stability	3.31	0.91	0.00	0.05 *	0.05 *	−0.01	0.09 *	0.13 *	0.27 *	0.01	*0.67*			
10.	Age (in years)	40.79	11.12	−0.36 *	0.03	−0.16 *	−0.18 *	−0.01	0.20 *	−0.05 *	0.06 *	0.05 *			
11.	Sex (0 = women, 1 = men)	0.47	0.50	0.06 *	0.03	0.20 *	−0.07 *	−0.07 *	−0.14 *	−0.09 *	−0.14 *	0.17 *	−0.04 *		
12.	Job type (0 = blue-collar, 1 = white-collar)	0.68	0.47	0.20 *	0.24 *	0.18 *	0.33 *	0.13 *	−0.04 *	0.12 *	0.07 *	0.01	−0.07 *	0.23 *	
13.	Years of education	13.88	2.33	0.36 *	0.38 *	0.49 *	0.54 *	0.16 *	−00.15 *	0.06 *	−0.02	0.02	−0.22 *	0.04 *	0.36 *

Note: Results are based on 50 multiple imputed datasets. Omega hierarchical and WLE reliabilities are in the diagonal. * *p* < 0.05.

**Table 2:** Cox regression models for intelligence, competence, and personality.

	Predictors	Model 1				Model 2			
		*B*	(*SE*)	OR	FMI	*B*	(*SE*)	OR	FMI
	*Intelligence*								
1.	Fluid intelligence	0.14 *	(0.03)	1.15	0.05	0.03	(0.04)	1.03	0.06
2.	Crystallized intelligence	0.06 *	(0.03)	1.06	0.11	0.08 *	(0.04)	1.09	0.12
3.	Age (in years)					−0.02 *	(0.00)	0.98	0.02
4.	Years in education					0.03 *	(0.01)	1.03	0.02
5.	Job type					0.14 *	(0.08)	1.15	0.06
	*R*^2^	0.02				0.06			
	BIC	16,553				13,027			
	*Competence*								
1.	Mathematical competence	0.06	(0.05)	1.06	0.41	0.04	(0.05)	1.04	0.40
2.	Reading competence	0.13 *	(0.05)	1.14	0.40	0.07 ^+^	(0.05)	1.07	0.38
3.	Age (in years)					−0.02 *	(0.00)	0.98	0.00
4.	Years in education					0.02 ^+^	(0.02)	1.02	0.06
5.	Job type					0.15 *	(0.08)	1.16	0.07
	*R*^2^	0.02				0.05			
	BIC	16,556				16,507			
	*Personality*								
1.	Openness	0.01	(0.03)	1.02	0.08	−0.02	(0.03)	0.98	0.08
2.	Conscientiousness	−0.04	(0.03)	0.96	0.05	0.03	(0.03)	1.03	0.07
3.	Extraversion	0.05 ^+^	(0.03)	1.05	0.11	0.02	(0.03)	1.02	0.10
4.	Agreeableness	0.00	(0.03)	1.00	0.07	0.00	(0.03)	1.00	0.08
5.	Emotional stability	0.07 *	(0.03)	1.07	0.07	0.09 *	(0.03)	1.09	0.08
6.	Age (in years)					−0.02 *	(0.00)	0.98	0.01
7.	Years in education					0.05 *	(0.01)	1.05	0.02
8.	Job type					0.16 *	(0.08)	1.18	0.07
	*R*^2^	0.01				0.06			
	BIC	16,603				16,524			

*Note*. Intelligence, competence, and personality scores were *z*-standardized. Stratified by sex resulting in different baseline hazard functions but constant coefficients across strata. *B* = Unstandardized regression weight, *SE* = Standard error for *B*, OR = Odds ratio, FMI = Fraction of missing information (i.e., the proportion of the total sampling variance due to missing data), BIC = Bayesian information criterion [49]. Based upon 50 imputation samples. * *p* < 0.05; ^+^ *p* < 0.10.

**Table 3:** Omnibus Cox regression models.

	Predictors	Model 1				Model 2			
		*B*	(*SE*)	OR	FMI	*B*	(*SE*)	OR	FMI
1.	Fluid intelligence	0.11 *	(0.04)	1.12	0.09	0.02	(0.04)	1.02	0.12
2.	Crystallized intelligence	0.02	(0.04)	1.02	0.17	0.08 *	(0.04)	1.08	0.18
3.	Mathematical competence	0.02	(0.06)	1.02	0.42	0.03	(0.06)	1.03	0.42
4.	Reading competence	0.10 *	(0.05)	1.11	0.41	0.05	(0.05)	1.05	0.41
5.	Openness	0.02	(0.03)	0.98	0.08	−0.04	(0.03)	0.96	0.08
6.	Conscientiousness	0.02	(0.03)	1.02	0.07	0.06 *	(0.04)	1.06	0.08
7.	Extraversion	0.04	(0.03)	1.04	0.11	0.02	(0.03)	1.02	0.12
8.	Agreeableness	0.01	(0.03)	1.01	0.07	0.01	(0.03)	1.01	0.08
9.	Emotional stability	0.07 *	(0.03)	1.07	0.07	0.08 *	(0.03)	1.09	0.08
10.	Age (in years)					−0.02 *	(0.00)	0.98	0.04
11.	Years in education					0.02 ^+^	(0.02)	1.02	0.06
12.	Job type (0 = blue-collar, 1 = white-collar)					0.13 ^+^	(0.08)	1.14	0.07
	*R*^2^	0.03				0.07			
	BIC	16,581				16,534			

*Note*. Intelligence, competence, and personality scores were *z*-standardized. Stratified by sex resulting in different baseline hazard functions but constant coefficients across strata. *B* = Unstandardized regression weight, *SE* = Standard error for *B*, OR = Odds ratio, FMI = Fraction of missing information (i.e., the proportion of the total sampling variance due to missing data), BIC = Bayesian information criterion [49]. Based upon 50 imputation samples. * *p* < 0.05; ^+^ *p* < 0.10.
